# Severe Cognitive Impairment in Trauma-Affected Refugees—Exploring the Impact of Traumatic Brain Injury

**DOI:** 10.3390/jcm13175096

**Published:** 2024-08-28

**Authors:** Linda Nordin, Søren Kit Bothe, Sean Perrin, Ia Rorsman

**Affiliations:** 1 DIGNITY—Danish Institute Against Torture, 2100 Copenhagen, Denmark; ln@dignity.dk (L.N.); sobo@dignity.dk (S.K.B.); 2 Department of Psychology, Lund University, 221-00 Lund, Sweden; 3 Department of Psychology, University of Copenhagen, 2100 Copenhagen, Denmark; 4 Department of Neurology and Rehabilitation Medicine, Skåne University Hospital, 221-85 Lund, Sweden; ia.rorsman@skane.se; 5 Department of Clinical Sciences Neurology, Lund University, 221-85 Lund, Sweden

**Keywords:** refugees, cognitive impairment, SDMT, TBI, head injury, PTSD

## Abstract

**Background/Objectives**: This study explores the relationship between cognitive performance measured by the Symbol Digit Modality Test (SDMT) and the severity of self-reported head injury, traumatic brain injury (TBI), post-traumatic stress disorder (PTSD), depression, pain, and psychosocial dysfunction in a population of trauma-affected refugees. Refugees, especially those who have been subjected to torture, often face various difficulties, such as PTSD, depression and somatic disturbances (e.g., pain), which can significantly impact their day-to-day functioning. **Methods**: Participants included 141 adult refugees (38% women) with a mean age of 45.4 years (SD = 9.4) and 9.7 years (SD = 4.9) of education who were referred for treatment of post-traumatic distress to DIGNITY, Danish Institute Against Torture. Participants completed standardized self-report measures of PTSD, anxiety, depression, pain, and health-related disability and measures of trauma history, physical injuries including head injury and loss of consciousness, and the SDMT, a quick standardized performance-based measure of cognitive impairment. **Results**: Eighty-eight percent of participants evidenced signs of substantial cognitive impairment as indexed by lower SDMT scores. Those with a self-reported history of TBI, marked by loss of consciousness, exhibited lower SDMT scores and higher health-related disabilities. Severity of PTSD, depression, anxiety, and pain were highly correlated with lower SDMT scores. TBI history was not significantly associated with the severity of PTSD, depression, anxiety, or pain, suggesting a complex interplay among these factors. **Conclusions**: Cognitive impairments are prevalent in trauma-affected refugees, interacting with symptoms of post-traumatic stress and pain, likely explaining the high disability levels in this population. Further research should employ a broader range of cognitive measures and detailed investigations of head injury/TBI experiences to investigate their impact on overall functioning, treatment response, and longer-term outcomes. This study adds to a small but growing body of studies documenting cognitive impairments in trauma-affected refugees, highlighting the importance of addressing cognitive impairments in treatment for trauma-affected refugees, particularly those with histories of torture and TBI. Clinicians working with trauma-affected refugees should consider the assessment of cognitive difficulties as part of comprehensive care planning.

## 1. Introduction

Refugees, especially those subjected to torture, often experience many difficulties, including post-traumatic stress disorder (PTSD), depression, and somatic disturbances (e.g., pain) that, together, interfere with day-to-day functioning [1,2] These impairments are likely to be the result of many factors, including decline in cognitive functioning, a common finding in non-refugee populations with PTSD and depression [3,4]. Studies of trauma-exposed individuals have found that severity of PTSD and depression are associated with cognitive deficits in attentional control, task-shifting, inhibition, information processing speed, and working- and long-term memory [5,6].

Additionally, cognitive impairments are also common among individuals whose traumatic exposure involved a head injury or other experiences capable of producing brain injury (e.g., suffocation, inhalation of toxic substances, rapid acceleration of the head), loss of consciousness (LOC), and/or post-traumatic amnesia [4,7,8]. Reports of LOC and/or post-traumatic amnesia suggest that a traumatic brain injury (TBI) has occurred [9]. TBI in refugees frequently occurs under difficult conditions before or during flight from their home countries. These injuries often result from torture, other organized violence, or war [10]. Furthermore, TBI has been associated with an increased risk of developing PTSD, and the cognitive impairments associated with PTSD are hard to distinguish from TBI [11].

Studies on combat veterans find that those with PTSD/depression and a TBI have greater impairments in cognitive functioning and poorer functional outcomes than those with TBIs or PTSD/depression alone [12], suggesting an interactional effect between PTSD and TBI on cognition [13]. There is preliminary evidence of a complex relationship between trauma, head injury/TBI, cognitive impairments, and PTSD in trauma-affected refugees. Several studies have described a high prevalence of self-reported TBI in refugee populations [14,15,16], but, to date, only three studies have examined the relationship between TBI and PTSD in refugees. The three studies find that a history of TBI is strongly associated with higher levels of depression and health complaints, including pain, with those having both TBI and PTSD showing the highest levels of overall disability. The other two studies found a history of TBI to be strongly associated with the severity of PTSD [16,17], whereas one did not [15]. In investigating the relationship between PTSD and cognition, Kivling-Bodén et al. (2003) found that PTSD severity was associated with alterations in fluid intelligence and episodic memory in civilian refugees from the war in Yugoslavia [18]. Yehuda et al. (2003) observed lower scores on a test of verbal learning in Holocaust survivors compared with trauma-exposed controls [19]. In a study of over 300 Congolese refugees, PTSD severity was found to be significantly associated with reductions in working memory and executive and psychosocial functioning [20].

### Aim of the Study

To date, no study has reported on the relationship between all three factors simultaneously: TBI, symptom severity in PTSD, and cognitive functioning in refugee populations. The present study aims to address this gap in the literature in a large sample of tortured refugees seeking treatment for post-traumatic distress. Based on the available literature, we anticipated that the refugees would show evidence of broad cognitive impairments rather than localized cognitive deficits, i.e., suggestive of an injury to specific brain regions. Thus, we chose the Symbol Digit Modality Test (SDMT) [21], a clinician-administered test of associative working memory, visual scanning, attention, and information processing speed that can be administered in less than five minutes. We expected that that the severity of cognitive impairments, as measured with SDMT, would be significantly correlated with severity of PTSD, depression, anxiety, pain, and health-related disability. We also anticipated that refugees with a history of TBI, defined by a history of head injury with loss of consciousness, would demonstrate additional cognitive impairment and more severe PTSD, depression, anxiety, pain, and health-related disability than those without TBI.

## 2. Materials and Methods

### 2.1. Setting and Population

The Danish Institute Against Torture (DIGNITY) operates a specialist outpatient clinic in Copenhagen, providing treatment targeting PTSD, depression, and somatic complaints in refugees exposed to torture and/or other organized violence. Patients included 141 adult refugees (38% women) referred to DIGNITY 2012–2014 and who were screened with the SDMT pre-treatment. The main countries of origin included Iraq (36%), Iran (18%), Lebanon (6%), Bosnia (5%), Afghanistan (4%), Somalia (4%), and Syria (2%). The patients had been residents in Denmark between 1 and 29 years. The patients had an average age of 45.4 years (SD = 9.4) and had been in school an average of 9.7 years (SD = 4.9) (please see previous publication for more detailed description of patients at DIGNITY) [22]. A large part of the population used antidepressants and sleep medication.

Inclusion criteria for the treatment were (1) 18 years or older; (2) arriving to Denmark as a refugee; (3) exposure to torture or organized violence; (4) permanent right to remain (asylum) in Denmark; (5) the ability to finance transportation to the clinic; (6) the presence of both primary psychiatric and somatic symptoms requiring treatment; (7) no current alcohol or drug-dependency; and (8) not presently suffering from psychosis.

### 2.2. Design and Procedures

The study was cross-sectional. At pre-treatment, all patients completed standardized self-report measures of mental/physical health and cognitive screening with the SDMT. Approximately half of the patients also completed Part 3 of the Harvard Trauma Questionnaire (HTQ) [23], which screens for possible head traumas, at pre-treatment. Whenever possible, the self-report measures were in the participant’s preferred language, and, where necessary, an interpreter assisted in completing the measures. All self-report measures used in this study have been found to be valid for their intended purposes and for use with refugees [24,25,26]. SDMT was chosen as a suitable screening tool to capture reductions in processing speed, attention, and working memory associated with TBI [27] and as a measure of general cognitive impairment and decline [28].

All patients gave informed consent to participate in the study. The Danish Data Protection Agency and the Danish Patient Safety Authority approved this research. Due to the nature of this research, the data will not be shared publicly, so supporting data are not available.

### 2.3. Measures

Traumatic exposure, possible brain injury, and PTSD were assessed using Parts 1, 3, and 4 of the Harvard Trauma Questionnaire (HTQ) [23]. Part 1 of the HTQ assesses (lifetime) exposure to 46 different types of traumatic events. Part 3 (5 items) measures history of possible brain injury, either through head injury or experiences that increase the risk of brain damage (e.g., suffocation, near-drowning, prolonged starvation) and loss of consciousness during a possible head trauma. In the present study, a traumatic brain injury (TBI) was defined as the occurrence of an experience capable of producing a brain injury, as assessed by Part 3 of the HTQ, including loss of consciousness, which would be a head injury event plus associated loss of consciousness. Part 4 (16 items) assesses the symptoms of PTSD as described in the 4th Revised Edition of the Diagnostic and Statistical Manual of Mental Disorders, where patients rate how much each symptom has troubled them over the past week (1 = not at all, 4 = extremely). The recommended cut-off score for a DSM-IV PTSD diagnosis is 2.5 [23].

Anxiety and Depression were assessed using the 25-item Hopkins Symptom Checklist (HSCL-25). Patients evaluate the extent to which each symptom bothered them during the past week (1 = not at all, 4 = extremely). A total score is computed based on the mean rating for all 25 items, as well as item means for anxiety (10 items) and depression (15 items). Suggested cut-off scores for the depression and anxiety subscales are 1.75 [29].

Pain severity and pain-related interference were assessed using the 9-item version of the Brief Pain Inventory (BPI) [30]. A two-dimensional representation of the human body is then presented, and respondents shade in body areas where they experience pain; the total number of shaded areas is calculated. Items 3–6 assess the worst, least, average, and current pain intensity (0 = no pain, 10 = worst pain imaginable). Items 7–8 assess medication use and relief from pain when using the medication (0% = no relief, 100% = complete relief). Item 9 assesses interference from pain (0 = no interference, 10 = complete interference) in general activity, mood, mobility, work, relations with others, sleep, and enjoyment of life. Mean scores are computed for items assessing pain severity (4 items) and pain interference (7 items).

### 2.4. Screening Cognitive Performance

The Symbol Digit Modality Test (SDMT) [21] was used as a measure of impairment and cognitive performance. SDMT is a widely used measure for assessing processing speed but also taps into attention, working memory, and incidental learning [31,32]. Information processing speed is often referred to as a foundation necessary for learning, word retrieval, and executive functions [33] and is commonly affected even in mild or diffuse injuries to the brain [27]. In the written format provided, patients were given a coded key matching nine abstract symbols corresponding with numerical digits. Below the key was a random sequence of these abstract symbols, each with a blank space underneath for filling in the matching number. The score represents the number of correct substitutions within 90 s, with possible scores ranging from 0 and 110. The written format is considered relatively free from cultural bias and serves as a useful screening tool for individuals who are not fluent in the testing language [34]. The SDMT demonstrates good psychometric properties and has proven sensitive in detecting cognitive impairments and decline across various disorders, including PTSD [35], and in several non-English speaking countries [36,37,38,39]. Along with high validity and reliability, these studies have shown SDMT to be easily performed and carried out by non-trained healthcare staff. One advantage to more conventional screening tests is that it does not have ceiling effects but can be used to assess performance in both high- and low-functioning populations. Due to its psychometric qualities, including being correlated to employment and daily functioning, it is considered a core neuropsychological measure in neurology [40].

Health-Related Disability was assessed using the 36-item WHO Disability Assessment Schedule (WHODAS 2.0) [25]. The WHODAS 2.0 assesses the impact of physical and psychiatric difficulties across six domains (6 items per domain): understanding and communicating, mobility, self-care, getting along with others, life activities, and participation in society. For each item, patients rate their difficulties over the past 30 days (1 = none; 5 = extreme or cannot do). An overall disability score is then generated using an algorithm that weights individual items and converts the total to a 0–100 scale, where higher scores indicate greater impairments. While normative data are available, there are no agreed upon clinical cut-offs. A study of US combat veterans applying for PTSD-related disability benefits concluded that a scores exceeding 40 indicated clinically-significant functional impairment [41], placing the individual in the top 10% of those reporting health-related disability according to published norms.

### 2.5. Statistical Analysis

Statistical analyses were conducted using SPSS for Mac, version 23.0. The percentage of missing data at the variable level was minimal (0–2.9%) across all measures, except for WHODAS 2.0, where 10.3% and 15% of responses were missing for two items (on the self-care and getting along with people sub-scales). Little’s MCAR test revealed that the data were missing at random, permitting imputation at the item level using the expectation maximization algorithm. For all patients, the proportion scoring within the clinical range for cognitive impairments on the SDMT was calculated, along with the pairwise correlations between SDMT scores and the measures of PTSD, depression, anxiety, pain, and health-related disability. Multiple linear regression models investigated the association between dependent variable health-related disability and cognitive impairment, pain interference, PTSD, depression, and anxiety. In the subset of patients who were screened with Part 3 of the HTQ, those who reported a head injury with LOC were compared to those with a head injury without accompanied LOC on measures of cognitive performance (SDMT), PTSD (HTQ-4), anxiety and depression (HSCL-25), pain (BPI), and health-related disability (WHODAS 2.0) via one-way analysis of variance (ANOVA). SDMT raw scores were compared to age-related normative data [42], and cognitive impairment was defined by performances ≥ 2 standard deviations (SD) below normative means.

## 3. Results

All patients scored above clinical cut-offs for either PTSD, anxiety, or depression: 90% for PTSD (HTQ-4 M = 3.15, SD = 0.48), 97% for anxiety (HSCL-anxiety M = 3.01, SD = 0.56), and 98% for depression (HSCL-depression M = 3.01, SD = 0.52).

SDMT raw scores from patients and age-relevant norms [42] as comparative references are reported in the following: patients under 30 years of age (N = 7): M = 41 (SD = 12.06) and age-relevant norms: M = 58.2 (SD = 9.1); patients in the age range 30–55 (N = 109): M = 25.89 (SD = 12.99) and age-relevant norms: M = 53.2 (SD = 8.9); and patients over 55 years (N = 25): M = 21.24 (SD = 12.4); age-related norms M = 35.8 (SD = 9.6).

Across all ages, when compared to published norm: M = 50.3 (SD = 9.0), 88% (124 of 141 patients) scored ≥ 2 SD below published normative means, i.e., in the cognitively impaired range. Education did not yield significant differences in performance when patients with ≥ 8 years of education (M = 27.2, SD = 13.4) were compared to those with ≤8 years or unreported (M = 25.2, SD = 15.4).

Table 1 presents the pairwise correlations between total scores on the SDMT, HTQ-4, HSCL-25, BPI, and WHODAS 2.0. Table 2 presents a multiple linear regression showing the relationship between health-related disability and cognitive impairment, pain interference, PTSD, depression, and anxiety in three different models.

The results from Table 1 show significant negative correlations between cognitive impairment and PTSD (r = −0.44), depression (r = −0.40), anxiety (r = −0.34), pain severity (r = −0.27), pain interference (r = −0.39), and health-related disability (r = −0.46).

The multiple linear regression analysis in Table 2 shows that cognitive impairment significantly predicts health-related disability in Model 1. In Model 2, when pain interference is added, both cognitive impairment and pain interference significantly predict health-related disability. In Model 3, PTSD, depression, and anxiety are also significant predictors of health-related disability.

Of the 141 patients, 80 (56.7%) were also screened for possible head trauma using Part 3 of the HTQ. The decision to conduct the head trauma screening (HTQ) was based on the therapist allocated to the participant rather than on symptoms, criteria, or selection. Of the patients screened with HTQ-3, 78 (98%) reported a possible head trauma. Of these, 64 patients (82%) reported a possible head trauma with LOC (i.e., TBI) and 14 (18%) reported a possible head trauma without LOC (No-TBI). Reports of LOC suggest that a traumatic brain injury (TBI) has occurred [9]. Table 3 presents the means, standard deviations, *p*-values, and Cohen’s *d* for scores on the SDMT, HTQ, HSCL-25, BPI and WHODAS 2.0 (separately) for the TBI (N = 64) and no-TBI (N = 14) groups. Compared to the no-TBI group, patients in the TBI-group had significantly poorer cognitive performances (SDMT) and health-related disability (WHODAS 2.0), with the observed differences in the moderate range. No significant between-group differences were observed for the severity of PTSD (Part 4, HTQ), depression or anxiety (HSCL-25), or for pain severity and pain-related interference (BPI).

## 4. Discussion

This study addresses a gap in the literature regarding the relationship between cognitive impairment, head trauma/TBI, and post-traumatic distress in clinically referred refugees exposed to torture and other organized forms of violence. Based on results from the SDMT, using a brief measure of information processing speed that is widely used to screen individuals with suspected cognitive impairment and brain disease/injury, a large majority (88%) of the refugees performed at levels suggestive of significant cognitive impairment, i.e., more than two standard deviations below population means, corresponding to ≤2nd percentile, which is generally considered in the impaired range. These results may reflect both organic impairment (TBI) and the effects of overall symptom load given the high levels of PTSD, depression, and pain in this sample. The exceptionally low levels of cognitive performance on the SDMT found here may help explain the high levels of impairment in everyday functioning found on the WHODAS 2.0. From previous research, we know that lower scores on the SDMT are associated with increased difficulties maintaining employment [44] and carrying out everyday life-skills such as money management and even conventional use of a computer [45,46]. This is one of the first studies to suggest that lower scores on the SDMT are related to functional difficulties in traumatized (and tortured) refugees.

It is important to acknowledge that cultural background can affect performance on cognitive tests [47,48], including the SDMT (a time-based test), e.g., with a cultural weighting on thoroughness rather than speed, but the extent of this effect was neither anticipated nor previously observed. [49,50]. Similarly, one could speculate that the present results may be explained by level of education [28]. However, as reported previously by Nordin et al. (2019), the educational level in this population is not unusually low (M = 9.06, SD 5.28) [22]. Consequently, neither cultural differences nor degree of schooling seem to fully explain the severity of cognitive impairments found in this culturally diverse sample of traumatized refugees.

Refugees in this study with lower SDMT scores also had significantly higher levels of interference from pain and health-related disability. As expected, individuals with more severe symptoms of PTSD, depression, anxiety, and pain had the lowest performance on the SDMT. The study reveals that cognitive impairment, particularly together with PTSD, depression, anxiety, and pain, is a significant predictor of health-related disability among tortured and trauma-affected refugees. The regression analysis demonstrates that these factors, when considered together, account for a high variance (R^2^ = 0.61) in health-related disability. Thus, the present findings add to a small body of literature, suggesting that the high levels of distress and disability observed in tortured and trauma-affected refugees with PTSD, depression, and pain may be partly owed to cognitive impairments.

Consistent with previous studies of tortured refugees [17], the majority of those screened for head traumas in this study reported one or more possible TBIs. Previous research has revealed that combat veterans with a history of both TBI and PTSD have greater cognitive and psychosocial impairments than those with only PTSD or TBI [13]. No such comparisons were possible in this study, because nearly all the refugees scored above the clinical cut-off on the self-report measures of PTSD. Nevertheless, when compared to the small subset of refugees who reported a head trauma but no loss of consciousness, those reporting consequent loss of consciousness (i.e., TBI) showed significantly lower cognitive performances and higher health-related disability in this sample. Consequently, these findings align closely with studies of combat veterans where differences in information processing speed have been used to differentiate PTSD patients with and without a history of TBI [51], as well as individuals with a history of TBI from controls [52].

Contrary to expectation, patients defined with TBI did not differ from those with a history of head traumas without loss of consciousness in terms of severity of symptoms of PTSD, depression, anxiety, or pain. Still, lower scores on the SDMT were associated with greater severity in these symptom domains in the sample at large. Research on non-refugee, trauma-exposed populations indicates that a history of mild TBI, involving no or very brief loss of consciousness, is associated with greater PTSD severity [53], but the relationship is less clear in moderate to severe TBI [54]. The present findings suggest a complex interaction between TBI, cognitive impairment, and post-traumatic distress in tortured and trauma-affected refugees seeking treatment for PTSD and related difficulties. The uneven groups and low number of head trauma without loss of consciousness in this analysis might have contributed to a problem with power that can lead to a false negative result regarding PTSD, depression, anxiety, and pain. Additional studies involving larger samples sizes, comparison groups, and a more fine-grained assessment of probable TBI and cognitive impairments are essential to delineate these relationships.

While the present study benefitted from the use of standardized measures of cognitive impairments, psychiatric symptoms, and health-related disability in a large sample of trauma-affected refugees, certain methodological limits must be noted. Only one neuropsychological test was used to screen for cognitive impairment, and self-report questionnaires were used for the psychiatric, pain, and TBI variables. Data for this study were also drawn from patients referred to a highly specialized outpatient mental health clinic for tortured refugees, very few of whom did not have PTSD, depression, or a history of TBI. A large part of the patients reported the use of sleep medications and antidepressants. Future studies should use a non-medicated control group to investigate effects of such medications on SDMT for this population. The SDMT has been validated as a measure of cognitive impairment in several patient groups and across nations, but further validation studies with refugee populations with regards to the heterogeneity of culture, language, and education are needed. Future studies involving cognitive impairment in this population would benefit from using SDMT alongside other cross-culturally validated screening tools for validation purposes, as well as other psychometric tests to explore the range of possible cognitive impairments. Although the SDMT was administered to all patients entering treatment during the study period, Part 3 of the HTQ (assessing head trauma/TBI) was only administered to slightly more than 50 percent of the patients.

Despite these limitations, the present study provides important data on the level of cognitive functioning in tortured refugees with a history of TBIs and the relationship of such functioning on PTSD, depression, anxiety, pain, and health-related disability. The present findings also suggest that further studies are needed to determine whether treatments for post-traumatic distress and somatic complaints can produce improvements in cognitive impairments in refugees with and without a history of TBI or vice versa. Given the detrimental consequences on everyday functioning associated with even diffuse forms of cognitive impairments such as slowed information processing [44], our findings raise the need to attend to this aspect of functioning for complete and improved patient management.

## 5. Conclusions

A majority of tortured and trauma exposed, treatment-seeking refugees scored in the cognitively impaired range on a widely used screening measure for neurological dysfunction. Lower levels of cognitive performances were associated with higher levels of posttraumatic distress, pain, and disability. Refugees with a reported history of head injury and loss of consciousness had the lowest levels of cognitive performance. Clinicians working with trauma-affected refugees should consider routine screening for head injury, loss of consciousness, and cognitive impairments. Moreover, the present findings point to the need for incorporating cognitive rehabilitation in treatment with patients exposed to torture and trauma.

## Figures and Tables

**Table 1:** Pairwise correlations among the measures of cognitive impairment, PTSD, depression, anxiety, pain, and overall disability/participation for all participants (N = 141).

Measure	Correlation (r)						
	1	2	3	4	5	6	7
1. Cog. Impairment (SDMT)	-						
2. PTSD (HTQ)	−0.44 **	-					
3. Depression (HSCL-25)	−0.40 **	0.78 **	-				
4. Anxiety (HSCL-25)	−0.34 **	0.60 **	0.62 **	-			
5. Pain Severity (BPI)	−0.27 **	0.29 **	0.19 *	0.25 **	-		
6. Pain Interference (BPI)	−0.39 **	0.45 **	0.37 **	0.44 **	0.68 **	-	
7. Health-related disability (WHODAS 2.0)	−0.46 **	0.70 **	0.67 **	0.63 **	0.33 **	0.57 **	-

Note: SDMT = Symbol Digit Modality Test; HTQ = Harvard Trauma Questionnaire part 4; HSCL-25 = Hopkins Symptom Checklist-25; BPI = Brief Pain Inventory; WHODAS 2.0 = World Health Organization Disability Assessment Schedule-2.0. ** *p* < 0.01 (2-tailed); * *p* < 0.05 (2-tailed).

**Table 2:** Summary of hierarchical regression analysis for health-related disability (WHO-DAS 2.0).

	Model 1			Model 2			Model 3		
Variable	B	SE B	β	B	SE B	β	B	SE B	β
Cognitive Impairment (SDMT)	−0.60	0.10	−0.46 **	−0.32	0.10	−0.24 **			
Pain Interference (BPI)				3.75	0.60	0.48 **			
PTSD (HTQ)							2.49	0.49	0.32 **
Depression (HSCL-25)							13.09	3.49	0.35 **
Anxiety (HSCL-25)							9.48	3.02	0.28 **
R2	0.21			0.38			0.61		
F for change in R2	33.8 **			37.6 **			65.0 **		

Note: ** *p* < 0.01.

**Table 3:** Means, standard deviations, and results of one-way ANOVA for refugees with a history of head trauma, with and without a loss of consciousness (LOC).

Measure	Head Trauma + LOC M (SD)	Head Trauma + No LOC M (SD)	F (DF)	Cohen’s d
Cognitive Impairment (SDMT)	26.34 (12.95)	34.5 (15.32)	4.26 (1.76) *	0.61
PTSD (HTQ)	3.13 (0.47)	2.91 (0.51)	2.37 (1.76)	
Depression (HSCL-25)	2.99 (0.55)	2.87 (0.53)	0.52 (1.76)	
Anxiety (HSCL-25)	3.01 (0.54)	2.79 (0.60)	1.87 (1.76)	
Pain Severity (BPI)	5.96 (2.29)	5.60 (1.95)	0.28 (1.74)	
Pain Interference (BPI)	6.71 (2.24)	5.97 (2.40)	1.15 (1.73)	
Health Related Disability (WHODAS 2.0)	65.81 (18.37)	54.97 (14.61)	4.11 (1.71) *	0.61

Note: * *p* < 0.05. SDMT = Symbol Digit Modality Test; HTQ4 = Harvard Trauma Questionnaire; HSCL-25 = Hopkins Symptom Checklist-25; BPI = Brief Pain Inventory; WHODAS 2.0 = World Health Organization Disability Assessment Schedule-2.0. Results based on 1000 bootstrap samples. Portions of the data reported in this table were presented in earlier works by the first author [43].

## Data Availability

Due to ethical reasons, the data will not be shared publicly, supporting data are therefore not available.

## References

[1] Fazel M., Wheeler J., Danesh J. (2005). Prevalence of serious mental disorder in 7000 refugees resettled in western countries: A systematic review. Lancet.

[2] Steel Z., Chey T., Silove D., Marnane C., Bryant R.A., van Ommeren M. (2009). Association of Torture and Other Potentially Traumatic Events with Mental Health Outcomes Among Populations Exposed to Mass Conflict and Displacement: A Systematic Review and Meta-analysis. JAMA.

[3] Geuze E., Vermetten E., de Kloet C.S., Hijman R., Westenberg H.G.M. (2009). Neuropsychological performance is related to current social and occupational functioning in veterans with posttraumatic stress disorder. Depress. Anxiety.

[4] Wrocklage K.M., Schweinsburg B.C., Krystal J.H., Trejo M., Roy A., Weisser V., Moore T.M., Southwick S.M., Scott J.C. (2016). Neuropsychological Functioning in Veterans with Posttraumatic Stress Disorder: Associations with Performance Validity, Comorbidities, and Functional Outcomes. J. Int. Neuropsychol. Soc..

[5] Parlar M., Densmore M., Hall G.B., Frewen P.A., Lanius R.A., McKinnon M.C. (2017). Relation between patterns of intrinsic network connectivity, cognitive functioning, and symptom presentation in trauma-exposed patients with major depressive disorder. Brain Behav..

[6] Vasterling J.J., Jacob S.N., Rasmusson A. (2018). Traumatic Brain Injury and Posttraumatic Stress Disorder: Conceptual, Diagnostic, and Therapeutic Considerations in the Context of Co-Occurrence. J. Neuropsychiatry Clin. Neurosci..

[7] Bradley L., Tawfiq N. (2006). The physical and psychological effects of torture in Kurds seeking asylum in the United Kingdom. Torture Q. J. Rehabil. Torture Vict. Prev. Torture.

[8] Keatley E., Ashman T., Im B., Rasmussen A. (2013). Self-Reported Head Injury Among Refugee Survivors of Torture. J. Head. Trauma Rehabil..

[9] Prince C., Bruhns M. (2017). Evaluation and Treatment of Mild Traumatic Brain Injury: The Role of Neuropsychology. Brain Sci..

[10] McPherson J.I. (2019). Traumatic brain injury among refugees and asylum seekers. Disabil. Rehabil..

[11] Cicerone K.D., Langenbahn D.M., Braden C., Malec J.F., Kalmar K., Fraas M., Ashman T. (2011). Evidence-Based Cognitive Rehabilitation: Updated Review of the Literature From 2003 Through 2008. Arch. Phys. Med. Rehabil..

[12] Polusny M.A., Kehle S.M., Nelson N.W., Erbes C.R., Arbisi P.A., Thuras P. (2011). Longitudinal Effects of Mild Traumatic Brain Injury and Posttraumatic Stress Disorder Comorbidity on Postdeployment Outcomes in National Guard Soldiers Deployed to Iraq. Arch. Gen. Psychiatry.

[13] Kaup A.R., Toomey R., Bangen K.J., Delano-Wood L., Yaffe K., Panizzon M.S., Lyons M.J., Franz C.E., Kremen W.S. (2019). Interactive Effect of Traumatic Brain Injury and Psychiatric Symptoms on Cognition among Late Middle-Aged Men: Findings from the Vietnam Era Twin Study of Aging. J. Neurotrauma.

[14] Doherty S.M., Craig R., Gardani M., McMillan T.M. (2016). Head injury in asylum seekers and refugees referred with psychological trauma. Glob. Ment. Health.

[15] Keatley E., d’Alfonso A., Abeare C., Keller A., Bertelsen N.S. (2015). Health Outcomes of Traumatic Brain Injury Among Refugee Survivors of Torture. J. Head. Trauma. Rehabil..

[16] Mollica R.F., Henderson D.C., Tor S. (2002). Psychiatric effects of traumatic brain injury events in Cambodian survivors of mass violence. Br. J. Psychiatry.

[17] Mollica R.F., Chernoff M.C., Berthold S.M., Lavelle J., Lyoo I.K., Renshaw P. (2014). The Mental Health Sequelae of Traumatic Head Injury in South Vietnamese Ex-Political Detainees Who Survived Torture. Compr. Psychiatry.

[18] Kivling-Bodén G., Sundbom E. (2003). Cognitive abilities related to post-traumatic symptoms among refugees from the former Yugoslavia in psychiatric treatment. Nord. J. Psychiatry.

[19] Golier J.A., Yehuda R., Lupien S.J., Harvey P.D. (2003). Memory for trauma-related information in Holocaust survivors with PTSD. Psychiatry Res..

[20] Ainamani H.E., Elbert T., Olema D.K., Hecker T. (2017). PTSD symptom severity relates to cognitive and psycho-social dysfunctioning—A study with Congolese refugees in Uganda. Eur. J. Psychotraumatol..

[21] Smith A. (1982). Symbol Digit Modalities Test.

[22] Nordin L., Perrin S. (2019). Pain and posttraumatic stress disorder in refugees who survived torture: The role of pain catastrophizing and trauma-related beliefs. Eur. J. Pain..

[23] Mollica R.F., Caspi-Yavin Y., Bollini P., Truong T., Tor S., Lavelle J. (1992). The Harvard Trauma Questionnaire: Validating a Cross-Cultural Instrument for Measuring Torture, Trauma, and Posttraumatic Stress Disorder in Indochinese Refugees. J. Nerv. Ment. Dis..

[24] Friis Jørgensen S., Auning-Hansen M.A., Elklit A. (2017). Can disability predict treatment outcome among traumatized refugees?. Torture J..

[25] Üstün T.B., Chatterji S., Kostanjsek N., Rehm J., Kennedy C., Epping-Jordan J., Saxena S., von Korff M., Pull C. (2010). Developing the World Health Organization Disability Assessment Schedule 2.0. Bull. World Health Organ..

[26] Wind T.R., van der Aa N., de la Rie S., Knipscheer J. (2017). The assessment of psychopathology among traumatized refugees: Measurement invariance of the Harvard Trauma Questionnaire and the Hopkins Symptom Checklist-25 across five linguistic groups. Eur. J. Psychotraumatology.

[27] Howieson D.B., Lezak M.D. (2012). The neuropsychological evaluation. Clinical Manual of Neuropsychiatry.

[28] Strober L., DeLuca J., Benedict R.H., Jacobs A., Cohen J.A., Chiaravalloti N., Hudson L.D., Rudick R.A., LaRocca N.G. (2019). Multiple Sclerosis Outcome Assessments Consortium (MSOAC) Symbol Digit Modalities Test: A valid clinical trial endpoint for measuring cognition in multiple sclerosis. Mult. Scler. J..

[29] Lavik N.J., Hauff E., Solberg Ø., Laake P. (1999). The use of self-reports in psychiatric studies of traumatized refugees: Validation and analysis of HSCL-25. Nord. J. Psychiatry.

[30] Cleeland C.S., Ryan K.M. (1994). Pain assessment: Global use of the Brief Pain Inventory. Ann. Acad. Med. Singap..

[31] Patel V.P., Walker L.A.S., Feinstein A. (2017). Deconstructing the symbol digit modalities test in multiple sclerosis: The role of memory. Mult. Scler. Relat. Disord..

[32] Sandry J., Simonet D.V., Brandstadter R., Kriege S., Katz Sand I., Graney R.A., Buchanan A.V., Lall S., Sumowski J.F. (2021). The Symbol Digit Modalities Test (SDMT) is sensitive but non-specific in MS: Lexical access speed, memory, and information processing speed independently contribute to SDMT performance. Mult. Scler. Relat. Disord..

[33] Costa S.L., Genova H.M., DeLuca J., Chiaravalloti N.D. (2017). Information processing speed in multiple sclerosis: Past, present, and future. Mult. Scler. J..

[34] O’Bryant S.E., Humphreys J.D., Bauer L., McCaffrey R.J., Hilsabeck R.C. (2007). The Influence of Ethnicity on Symbol Digit Modalities Test Performance: An Analysis of a Multi-Ethnic College and Hepatitis C Patient Sample. Appl. Neuropsychol..

[35] Qureshi S.U., Long M.E., Bradshaw M.R., Pyne J.M., Magruder K.M., Kimbrell T., Hudson T.J., Jawaid A., Schulz P.E., Kunik M.E. (2011). Does PTSD Impair Cognition Beyond the Effect of Trauma?. J. Neuropsychiatry Clin. Neurosci..

[36] Eshaghi A., Riyahi-Alam S., Roostaei T., Haeri G., Aghsaei A., Aidi M.R., Sahraian M.A. (2012). Validity and Reliability of a Persian Translation of the Minimal Assessment of Cognitive Function in Multiple Sclerosis (MACFIMS). Clin. Neuropsychol..

[37] Estiasari R., Fajrina Y., Lastri D.N., Melani S., Maharani K., Imran D., Pangeran D., Sitorus F. (2019). Validity and Reliability of Brief International Cognitive Assessment for Multiple Sclerosis (BICAMS) in Indonesia and the Correlation with Quality of Life. Neurol. Res. Int..

[38] Ozakbas S., Yigit P., Cinar B.P., Limoncu H., Kahraman T., Kösehasanoğulları G. (2017). The Turkish validation of the Brief International Cognitive Assessment for Multiple Sclerosis (BICAMS) battery. BMC Neurol..

[39] Spedo C.T., Frndak S.E., Marques V.D., Foss M.P., Pereira D.A., Carvalho L.D.F., Barreira A.A. (2015). Cross-cultural Adaptation, Reliability, and Validity of the BICAMS in Brazil. Clin. Neuropsychol..

[40] Benedict R.H., DeLuca J., Phillips G., LaRocca N., Hudson L.D., Rudick R., Multiple Sclerosis Outcome Assessments Consortium (2017). Validity of the Symbol Digit Modalities Test as a cognition performance outcome measure for multiple sclerosis. Mult. Scler. J..

[41] Marx B.P., Wolf E.J., Cornette M.M., Schnurr P.P., Rosen M.I., Friedman M.J., Keane T.M., Speroff T. (2015). Using the WHODAS 2.0 to Assess Functioning Among Veterans Seeking Compensation for Posttraumatic Stress Disorder. Psychiatr. Serv..

[42] Sheridan L.K., Fitzgerald H.E., Adams K.M., Nigg J.T., Martel M.M., Puttler L.I., Wong M.M., Zucker R.A. (2006). Normative Symbol Digit Modalities Test performance in a community-based sample. Arch. Clin. Neuropsychol..

[43] Nordin L. (2019). Posttraumatic Stress Reactions in Tortured Refugees: Relationship to Pain, Cognitive Impairments, and Negative Appraisals. Ph.D. Thesis.

[44] Honan C.A., Brown R.F., Batchelor J. (2015). Perceived Cognitive Difficulties and Cognitive Test Performance as Predictors of Employment Outcomes in People with Multiple Sclerosis. J. Int. Neuropsychol. Soc..

[45] Goverover Y., Chiaravalloti N., DeLuca J. (2016). Brief International Cognitive Assessment for Multiple Sclerosis (BICAMS) and performance of everyday life tasks: Actual Reality. Mult. Scler. J..

[46] Goverover Y., Haas S., DeLuca J. (2016). Money Management Activities in Persons with Multiple Sclerosis. Arch. Phys. Med. Rehabil..

[47] Ardila A. (2005). Cultural values underlying psychometric cognitive testing. Neuropsychol. Rev..

[48] Franzen S., Watermeyer T.J., Pomati S., Papma J.M., Nielsen T.R., Bekkhus-Wetterberg P., European Consortium on Cross-Cultural Neuropsychology (ECCroN) (2022). Cross-cultural neuropsychological assessment in Europe: Position statement of the European Consortium on Cross-Cultural Neuropsychology (ECCroN). Clin. Neuropsychol..

[49] Ryan J., Woods R.L., Britt C.J., Murray A.M., Shah R.C., Reid C.M., Storey E. (2020). Normative Data for the Symbol Digit Modalities Test in Older White Australians and Americans, African-Americans, and Hispanic/Latinos. J. Alzheimers Dis. Rep..

[50] Messinis L., Bakirtzis C., Kosmidis M.H., Economou A., Nasios G., Anyfantis E., Konitsiotis S., Ntoskou A., Peristeri E., Dardiotis E. (2021). Symbol digit modalities test: Greek normative data for the oral and written version and discriminative validity in patients with multiple sclerosis. Arch. Clin. Neuropsychol..

[51] Combs H.L., Berry D.T.R., Pape T., Babcock-Parziale J., Smith B., Schleenbaker R., Shandera-Ochsner A., Harp J.P., High W.M. (2015). The Effects of Mild Traumatic Brain Injury, Post-Traumatic Stress Disorder, and Combined Mild Traumatic Brain Injury/Post-Traumatic Stress Disorder on Returning Veterans. J. Neurotrauma.

[52] Dymowski A.R., Owens J.A., Ponsford J.L., Willmott C. (2015). Speed of processing and strategic control of attention after traumatic brain injury. J. Clin. Exp. Neuropsychol..

[53] Dolan S., Martindale S., Robinson J., Kimbrel N.A., Meyer E.C., Kruse M.I., Morissette S.B., Young K.A., Gulliver S.B. (2012). Neuropsychological Sequelae of PTSD and TBI Following War Deployment among OEF/OIF Veterans. Neuropsychol. Rev..

[54] Mayou R.A., Black J., Bryant B. (2000). Unconsciousness, amnesia and psychiatric symptoms following road traffic accident injury. Br. J. Psychiatry.

